# Ulnocarpal stabilization in the management of comminuted fractures distal end radius

**DOI:** 10.4103/0019-5413.44631

**Published:** 2009

**Authors:** Dinesh K Gupta, Gaurav Kumar

**Affiliations:** Department of Orthopaedics, MLB Medical College, Jhansi Orthopaedic Hospital and Research Centre, Jhansi, India; 1 Department of Orthopaedics, LLR Medical College, Meerut, India

**Keywords:** Comminuted fracture of the distal end radius, percutaneous pinning, stabilization of ulnocarpal articulation

## Abstract

**Background:**

Malunion due to progressive radial collapse during healing is a common complication following comminuted fracture distal radius treated by conventional methods. Many treatment modalities have been described with their own merits and demerits. Stabilization of ulnocarpal articulation is an effective method to prevent radial collapse during healing, and hence this study.

**Materials and Methods:**

A prospective study of 200 patients of comminuted intraarticular fracture lower end radius between 20–75 years of age irrespective of sex, treated by closed reduction and percutaneous stabilization of ulnocarpal articulation and a well-molded above elbow POP cast for 6 weeks has been presented. Patients were evaluated at 1 year follow-up and functionally by Sarmiento's modification of Lindstrom criteria and Gartland and Werley's criteria.

**Results:**

Excellent to good results were seen in 92%, fair in 6% and poor in 2% of the cases. Complications observed were, pintract infection (*n* = 6), malunion (*n* = 6), subluxation of the inferior radioulnar joint (*n* = 4) Sudeck's osteodystrophy (*n* = 3) and post-traumatic arthritis of the wrist (*n* = 3).

**Conclusion:**

Percutaneous fixation by this technique is an effective method to maintain the reduction, prevent radial collapse during healing, and to maintain the stability of the distal radioulnar joint even when the fracture is grossly comminuted, intraarticular, or unstable.

## INTRODUCTION

Fractures of the lower end radius constitute 17% of all fractures and 75% of all forearm fractures.^1^ Closed reduction and cast immobilization has been the mainstay of treatment of these fractures, but malunion of fracture and subluxation or dislocation of distal radioulnar joint resulting in poor functional and cosmetic results is the usual outcome.^2^ The restoration of the anatomy correlates well with function. The residual deformity of wrist due to malunion of the fractures of the distal end radius and subluxation or dislocation of distal radioulnar joint which is unsightly and adversely affects the wrist motion and hand function by interfering with the mechanical advantage of the extrinsic hand musculature.^3^–^5^ It may cause pain, limitation of forearm motion, and decreased grip strength as a result of arthrosis of the radiocarpal and distal radioulnar joints,^6^–^8^ incongruity of the distal radioulnar joint,^6^,^9^ ulnocarpal impingement^1^,^10^ and carpal malalignment.^11^ To overcome these, Darrach^12^ described the excision of the distal end ulna in cases with disruption of distal radioulnar articulation with or without malunion of distal radius, but results are unpredictable; in many instances, it leads to weakness of grip as well as instability of the distal end ulna, and hence, there was a need to evolve more aggressive and effective fixation of these fractures. Many treatment modalities have been described with the aim of achieving and maintaining good anatomical and functional end results.

Various modalities of treatment available are closed reduction and POP cast immobilization, functional cast bracing, pin and plaster technique, rush rod fixation, external fixators, percutaneous pin fixation, arthroscopic reduction, open reduction, and internal fixation. Each procedure has its own merits and demerits.

Closed reduction and cast immobilization^13^,^14^ has been the mainstay of treatment, but collapse of the radius occur even in the face of cast immobilization.

Percutaneous pins to provide additional stability is one of the earliest forms of internal fixation.^15^–^17^ Depalma^17^ described ulnaradial pinning drilled at a 45° angle, 4 cm proximal to ulnar styloid, from ulna into radial styloid. Stein^18^ described the use of an additional dorsal pin, an additional 2- mm pin across the radioulnar joint was also used^19^, Kapandji^20^ described double intrafocal pining (2- mm) into the fracture surface, and Rayhack^21^ described ulnaradial pinning with fixation of distal radioulnar joint.

Bridging external fixators and ligamentotaxis indirectly reduce the impacted articular fragments and directly neutralizes the axial load. Several workers^22^,^23^ reported superior radiographic outcome as compared with cast treatment.

Ruch *et al*,^24^ Schnnur,^25^ and many others described ORIF of distal radial fractures. In gross comminution of articular segments, Bass,^26^ Rikli *et al*,^27^ and others recommended a combination of ORIF, external fixators, and K-wires to create an intact palmar buttress by using a plate and avert dorsal collapse by tensioning across it using an external fixator. Doi *et al*^28^ recommended arthoscopically guided reduction.

The biomechanical study by Graham^29^ reported that although the stabilization of distal fibula in ankle injuries plays a role, the construct in which ulna is engaged provide superior resistance to fracture displacement with the availability of intact cortical bones, i.e., ulna and carpals. The author thought that the role of stabilization of distal ulnocarpal articulation is important in managing comminuted fractures of lower end radius and studied the importance of distal radioulnar joint (DRUJ) and triangular fibrocartilage complex (TFCC) in realigning the anatomy of the wrist joint. The TFCC is the main stabilizer of DRUJ. In Colles' fracture, the breaking of the distal end of the radius, with its impaction and dorsal displacement, attenuates this fibrocartilage to a maximum degree because of the volar displacement of the ulnar head. Considering mainly the cortical nature and intactness of the distal ulna and carpals in these fractures, the present authors emphasized upon closed reduction and stabilization of the ulnocarpal joint, which indirectly helps to align the fracture fragments (ligamentotaxis) to maintain the radial length, the articulating surface of the distal radius, realigning the TFCC and, DRUJ and also preventing the redisplacement of fracture fragments and collapse during fracture healing, hence this study where we conducted a retrospective analysis of 200 patients of fracture of lower end radius treated by closed reduction and percutaneous stabilization of distal end of ulna.

## MATERIALS AND METHODS

This retrospective analysis was conducted in 200 patients with comminuted fracture of the lower end radius who could be followed up from January 2004 to December 2006. All the cases of comminuted fracture lower end radius were included in the study except those patients, who did not gave their consent; patients with more than 3 weeks duration of injury; patients in whom shaft ulna was not intact; patients in whom acceptable reduction could not be achieved; and those who were lost to follow-up.

The cases were grouped as per Melon's^30^ classification of intraarticular fractures distal radius.

**Figure 1:**
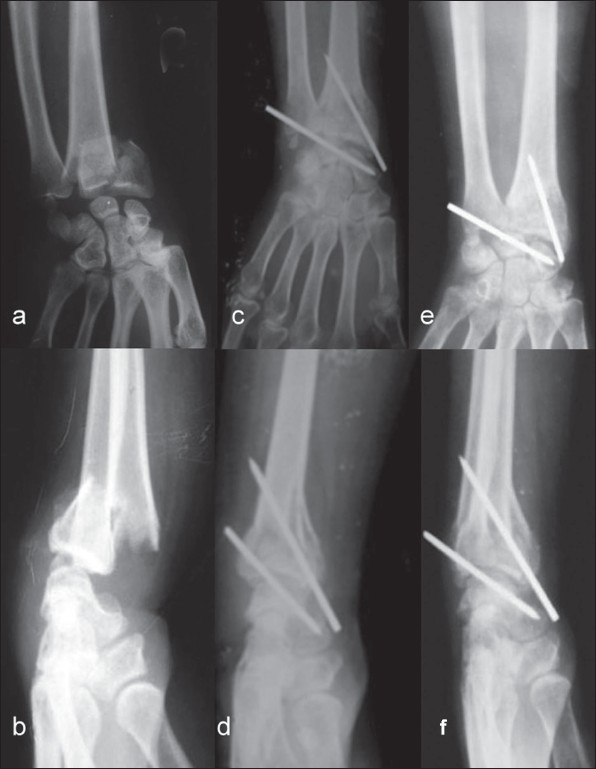
Pre operative anteroposterior and lateral (a, b) xray of the left wrist shows comminuted intraarticular fracture distal end radius (Melon's type-II) with fracture ulnar styloid. Immediate post operative and anterioposterior and lateral X-rays (c, d) and 6 weeks follow-up anteroposterior and lateral X-rays (e, f) shows evidence of union, maintenance of alignment of DRUJ, radial length, normal radial tilt.

The patients were treated by close reduction and percutaneous stabilization of ulnocarpal articulation under an image intensifier. The fracture was immobilized in a well-molded above elbow plaster of paris (POP) cast for 6 weeks. The patients were followed at 3 weeks interval up to 6 weeks and then at 6 weeks interval for 1 year. [Figure 1]

**Figure 2:**
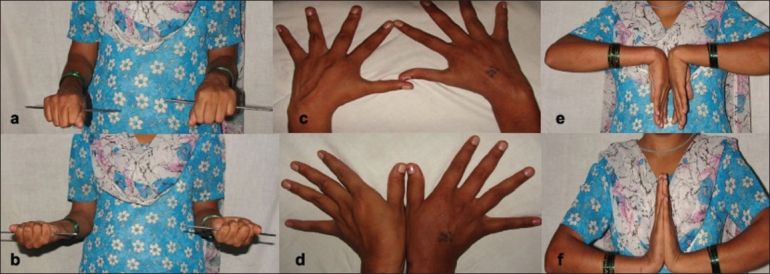
Follow-up clinical photograph of the same patient shows full range of pronation and supination (a, b) radial and ulnar deviation (c, d) palmar flexion and dorsiflexion (e, f).

Results were evaluated clinically and radiologically at 1-year follow-up [Figure 2] using the Sarmiento's modification of Lindstrom Criteria^31^ [Table 1] and the functional evaluation by the demerit point system of Gartland and Werley's^32^ with Sarmiento *et al* 's modification [Table 2].

**Table 1:** Sarmiento's modification of LindStrom Criteria (Anatomical evaluation)

	Residual deformity	Loss of palmar inclination	Radial shortening (mm)	Loss of radial deviation
Excellent	No or insignificant	0°	<3	<5°
Good	Slight	1–10°	3–6	5–9°
Fair	Moderate	11–14°	7–11	10–14°
Poor	Severe	Atleast 15°	Atleast 12	>14°

**Table 2:** Demerit point system of Gartland and Werley's with Sarmiento *et al.* modification (functional evaluation)

Result	Points
Residual deformity	
Prominent ulnar styloid	1
Residual dorsal tilt	2
Radial deviation of hand	2–3
Point range	0–3
Subjective evaluation	
Excellent – no pain, disability, or limitation of motion	0
Good – occasional pain, slight limitation of motion, no disability	2
Fair – occasional pain, some limitation of motion, feeling of weakness in wrist, no particular disability if careful, activities slightly restricted	4
Poor – pain, limitation of motion, disability, activities more or less markedly restricted	6
Point range	0–6
Objective evaluation*	
Loss of dorsiflexion	5
Loss of ulnar deviation	3
Loss of supination	2
Loss of palmar flexion	1
Loss of radial deviation	1
Loss of circumduction	1
Loss of pronation	2
Pain in distal radioulnar joint	1
Grip strength - 60% or less of opposite side (Using dynamometer)	1
Point range	0–5
Complications	
Arthritic change	
Minimum	1
Minimum with pain	3
Moderate	2
Moderate with pain	4
Severe	3
Severe with pain	5
Nerve complications (Median)	1–3
Poor finger functions due to cast	1–2
Point range	0–5
End result point ranges	
Excellent	0–2
Good	3–8
Fair	9–20
Poor	21 and above

* The objective evaluation is based on the following ranges of motion as being the minimum for normal function: dorsiflexion 45°; palmar flexion 30°; radial deviation 15°; ulnar deviation 15°; pronation 50°; supination 50°

Melon's classification of intraarticular fractures (subtype of universal classification)^30^

- Type I - undisplaced
- Type II - median column displacement (die-punch fracture)
- Type III - segmental radial shaft (butterfly fragment) component
- Type IV - transverse spilt of articular surfaces with rotational displacement

### Measurement of various angles

The various angles measured were as described

Palmar tilt (RT): This is measured in a true lateral X-ray of the wrist. A line perpendicular to the central axis of the radius is drawn through the dorsal rim of the distal radius. Another line joins the dorsal and ventral rim of the radius. The angle of palmar tilt is 0–22°

Radial length (RL): In true anteroposterior x-ray of the wrist, two lines are drawn perpendicular to the long axis of radius, one joining the tip of the radius styloid and the other joining the distal articular surface of the ulna. The distance between these two lines is called radial length and should be 11–12 mm.

Radial angulation (RA): In true AP X-ray of the wrist, a line perpendicular to the central axis of the radius is drawn. Another line joins the distal tip of the radial styloid and the ulnar corner of the ulnar fossa. The angle between lines 1 and 3 normally measures 16–28° (in true AP skiagram of wrist).

### Operative procedure

Close reduction is done by longitudinal traction, and direct pressure on displaced radial fragment depending upon displacement of fragments under general or regional anesthesia and the reduction was checked by an image intensifier. The goal is to approximate the palmar cortex. While maintaining the position and keeping forearm in 30° supination, the first K-wire (2 to 2.5 mm) is negotiated through the dorsomedial border of distal ulna about 1.5 cm proximal to ulnar styloid at 45° from long axis of ulna in distal and radial direction and 10–15° ventral direction into the adjacent carpal/carpals (lunate/scaphoid). Another K-wire is negotiated from the ventrolateral aspect of radial styloid at 45° with the long axis of radius 10–15° dorsally and medially so as to penetrate the medial cortex of proximal segment of radius, under the control of an image intensifier, and the position was checked in AP and lateral views. A well-molded above elbow POP cast is given. Postoperatively the limb is kept elevated, and active finger and shoulder exercise are started at the earliest. The K-wires and the POP are removed after 6 weeks, and active exercises of wrist and supination and pronation of forearm are started.

## RESULTS

A total of 192 patients with 200 comminuted fractures of distal end radius with an average age of 48 years (range 20–75 years) were included in this study. Of those, 182 cases were fresh (within 24 h of injury), nine were operated within 1 week, five cases were operated within 2 weeks, and four were operated within 3 weeks of injury. Fall on outstretched hand was the most common mode (n=168, 84%) of injury followed by road traffic accidents (n=30, 15%). Right wrist was involved in 108 patients, left in 76 patients, and bilateral involvement in 8 patients. Fifty-eight of our fractures were Melon's type-I, 129 fractures were Melon's type-II, nine were Melon's type-III, and four fractures were Melon's type-IV fractures. Results were assessed clinically and radiologically according to anatomical and functional criteria and were compared with the normal wrist. In the bilateral cases where the normal wrist was not available for comparison, the results were evaluated in relation to the documented normal values.

Anatomically 75% fractures had excellent restoration of anatomy, 17% fractures had good restoration of anatomy, 6.5% had fair, and 1.5% had poor restoration. Thus, 92% fractures had excellent to good alignment of fragments [Table 3].

**Table 3:** Anatomical evaluation Sarmiento's modification of LindStrom criteria

	Residual deformity (%)	Loss of palmar inclination (%)	Radial shortening (%)	Loss of radial deviation (%)	Mean (%)
Excellent	168 (84)	148 (74)	160 (80)	140 (70)	149 (74.5)
Good	24 (12)	40 (20)	32 (16)	32 (16)	35 (17.5)
Fair	6 (3)	8 (4)	6 (3)	24 (12)	13 (6.5)
Poor	2 (1)	4 (2)	2 (1)	4 (2)	3 (1.5)

Functionally, 79% fractures had excellent and 12% had good restoration of functions. Eighteen fractures (9%) could not regain normal or near-normal functions because of either poor reduction or lack of cooperation in exercise program. Poor function correlated with residual displacement and poor patient compliance [Table 4].

**Table 4:** Functional evaluation - Demerit point system of Gartland and Werley's

	Score	No. of cases (%)
Excellent	0–2	158 (79)
Good	3–8	24 (12)
Fair	9–20	14 (7)
Poor	21 and above	4 (2)

Thus, overall 92% fractures had excellent to good results, 6% fair, and 2% had poor results.

The complications encountered were pintract infection (n=6), malunion (n=6), subluxation of inferior radioulnar joint (n=4), Sudeck's osteodystrophy (n=3), post traumatic arthritis of wrist (n=3), penetration of vessels (n=2), joint stiffness (n=8), reduced grip strength (n=10) and paraesthesia in the distribution of superficial radial nerve (n=1).

## DISCUSSION

The ideal method of treatment of fracture of lower end radius is yet to emerge. Treatment modality should satisfy both functional and cosmetic results. Closed reduction and POP immobilization is still practiced in developing countries because of limited facilities. Its merits include no need for metal insertion, cost effectiveness, and less time consuming. High incidence of failure in grossly comminuted fractures and loss of reduction after successful reduction are the most common demerits. Conventional immobilization with POP often does not prevent early collapse and has high risk of malunion, joint stiffness, DRUJ instability, and painful wrist and is good only for (a) stable extraarticular fractures and (b) low demand elderly patients.^7^,^33^

Pin and plaster technique, percutaneous pinning of the distal fragment, external fixators devices, open reduction and internal fixation, and a combination of the above have been described to present collapse/loss of reduction, with their own advantages and disadvantages.

The complication of incorporating pins into the circumferential plaster has led to a reevaluation of pin and plaster technique.^34^ Chapman^35^ in a study reported no radial shortening in 44%, 1–5 mm of radial shortening in 30%, and more than 5 mm of radial shortening in 26%. Other complications reported were significant pin tract infection (20%), osteomyelitis (9%), iatrogenic fractures (9%), neurosensory complications (13.7%), and pin loosening (21%).

Munson and Gainor^36^ using percutaneous pinning of radial styloid fragment to opposite radial cortex reported restoration and conservation of radial angle in 87% and radial length in 92% of fractures but suggested that the technique is not good for elderly patients with thin cortices of osteoporotic bones. Percutaneous pinning of radius is biomechanically unsound, and the oblique orientation of pins is unable to prevent radial collapse. Hochwald and associates^37^ reported 9% extensor tendon injury, 32% superficial radial nerve injury, along with pin tract infection and pin migration as important complications of this technique.

Nonnenmacher and Kempfe^38^ described 90% satisfactory results using Kapandji's intrafocal pinning. David^39^ emphasized upon the tendency of distal fragment to displace in opposite direction, preventing the palmar cortex from reducing anatomically. The technique gives poor results with extensive comminution, osteoporosis, or intraarticular fractures. Thus, various methods of percutaneous pin fixation described till date have loss of reduction as one of the most common complications.^11^ External fixators can maintain the radial length and radial inclination by ligamentotaxis but cannot effectively maintain the palmar tilt of the distal articular surface.^40^ The thick, strong V-shaped volar ligaments have been shown to reach maximum tension when compared with the thinner Z-shaped dorsal ligaments. With longitudinal traction, this anatomic configuration predisposes the fracture to maintain dorsal tilt. Complications with the use of external fixators have been reported to be as high as 60% and include added risk of pin loosening, pin tract infection,^41^ Sudeck's osteodystrophy, radial sensory neuritis, and iatrogenic fracture through the pin site. Potential detrimental effects of over distraction include finger clawing, inability to make fist, delayed union, and residual stiffness. Several detailed studies^40^,^42^ have documented that external fixators alone may not be sufficiently rigid to prevent some degree of collapse and loss of palmar tilt during healing.

Open reduction and internal fixation is not frequently used because of technical problems in stabilizing multiple small cancellous fragments in an osteoporosed bone and has complications such as loss of fixation and other complications of open surgery. This method has limited indications, such as partial articular fractures, complex intraarticular fractures, and failed closed reduction.^25^

Combined ORIF and external fixators lead to extensive soft tissue stripping.^43^ Ruch^44^ compared the outcome of arthroscopy-assisted reduction, percutaneous pinning, and external fixation with fluoroscopy-assisted reduction, percutaneous pinning and external fixation and concluded that although arthroscopy provides superior visualization of articular surface and ulnar sided components of injury achieved a greater degree of supination, flexion, and extension. The fluoroscopic assisted reduction, external fixation permits some collapse during healing, which may retract from subsequent radiographic outcome. Radial shortening, knirk, and Jupiter congruity grades and DASH scores were similar for both groups.

Considering mainly the cortical nature of distal intact ulna and intact carpals in these fractures, we stabilize the ulnocarpal joint, which indirectly helps to align the fracture fragments (ligamentotaxis) to maintain the radial length, the articulating surfaces of the distal radius, realigning the TFCC and DRUJ and also preventing the collapse during fracture healing. A biomechanical study by Graham *et al.*^29^ on percutaneous pinning of distal radial fractures concluded that constructs in which ulna is engaged provide superior resistance to fracture displacement. Our aim in the present technique is to maintain the reduction achieved, to retain the preinjury anatomy to as near normal as possible. Ulnocarpal fixation after close reduction and restoration of anatomy helped in realigning the TFCC (a hole of 2–2.5 mm smooth K-Wire is of no consequence). DRUJ dysfunction (pain and limitation of forearm rotation) is a main cause of residual wrist disability.^45^ DRUJ instability (subluxation); intraarticular incongruity, and ulnocarpal abutment are the main factors for DRUJ dysfunction despite perfect anatomical reduction of distal radius. Thus, good alignment of ulnocarpal joint during reduction and healing is an important consideration to bring about good anatomical and functional outcome.

The procedure is of short duration and can be done under general, regional, or even local anesthesia. Also the removal of K-Wires can be done as an out patient procedure. The percutaneous fixation by this technique is an effective method to maintain the reduction and prevent collapse of radial fragments as well as to maintain the stability of DRUJ, even when fracture is grossly comminuted, intraarticular, or unstable. A study reported by Rayhack^21^ compared radial styloid pinning, radial styloid and posteromedial pinning, and transulnar pinning and concluded that two pin Clancey method was least stiff, followed by radial styloid pinning. The most stiff technique was that of transulnar pinning.

The present technique transfixes the two cortical bones, i.e., ulnar and carpals and stabilizes the ulnocarpal articulation, making it still more stiff; hence, the incidence of radial shortening of more than 6 mm was seen only in 4% of our fracturres when compared with 26% as reported by Chapman.^35^ Four out of 27 patients reported by Rayhack *et al*^21^ had pin breakage while rupture of extensor tendons/tendinitis (2%) and redisplacement of fracture fragments (14%) as reported by Nonnenmacher and Kempfe^38^ were not seen in any of the cases of this series.

## CONCLUSION

The percutaneous pinning by this technique is least invasive, yet an effective method to maintain the reduction and stability of distal radioulnar joint, and prevent collapse of radial fragments during healing, even when the fracture is grossly comminuted, intraarticular, or unstable. Overall excellent to good (92%) results in our study suggest that stabilizing lower end ulna with carpals, stabilizes the TFCC, which is the main stabilizer of DRUJ and maintains radial length, which is a crucial factor in regaining the functions of the wrist joint.
